# FDG-PET/CT in Lymphoma: Where Do We Go Now?

**DOI:** 10.3390/cancers13205222

**Published:** 2021-10-18

**Authors:** Yassine Al Tabaa, Clement Bailly, Salim Kanoun

**Affiliations:** 1 Scintidoc Nuclear Medicine Center, 25 rue de Clémentville, 34070 Montpellier, France; 2 CRCINA, INSERM, CNRS, Université d’Angers, Université de Nantes, 44093 Nantes, France; clement.bailly@chu-nantes.fr; 3 Nuclear Medicine Department, University Hospital, 44093 Nantes, France; 4 Nuclear Medicine Department, Institute Claudius Regaud, 31100 Toulouse, France; kanoun.salim@iuct-oncopole.fr; 5 Cancer Research Center of Toulouse (CRCT), Team 9, INSERM UMR 1037, 31400 Toulouse, France

**Keywords:** FDG-PET, lymphomas, Deauville 5PS

## Abstract

**Simple Summary:**

We focus on the role and current applications of 18F-fluorodeoxyglucose positron emission tomography combined with computed tomography in the management of lymphoma patients through improved staging or treatment response assessment or through early PET-driven therapeutic strategies, leading the way to a novel area of personalized medicine, optimizing disease control and toxicity. We discuss the potential future directions of innovative metabolic metrics that are being developed, notably to assess response to new immunotherapy regimens and to provide an improved prognostic factor for predicting patients’ survival. Finally, we present new radiopharmaceuticals developed following the identification of pathways or specific receptors in lymphomas, providing great opportunities for molecular imaging in treatment evaluation and management.

**Abstract:**

18F-fluorodeoxyglucose positron emission tomography combined with computed tomography (FDG-PET/CT) is an essential part of the management of patients with lymphoma at staging and response evaluation. Efforts to standardize PET acquisition and reporting, including the 5-point Deauville scale, have enabled PET to become a surrogate for treatment success or failure in common lymphoma subtypes. This review summarizes the key clinical-trial evidence that supports PET-directed personalized approaches in lymphoma but also points out the potential place of innovative PET/CT metrics or new radiopharmaceuticals in the future.

## 1. Introduction

Over the past several decades, positron emission tomography coupled with computed tomography using 18F-fluorodeoxyglucose (FDG-PET/CT), a tracer of glucose metabolism, has been considered as state-of-the-art imaging and provided the most significant improvement compared to conventional imaging in staging lymphoma [1,2]. At baseline, FDG-PET/CT evaluation offers a better staging than conventional CT especially for extra nodal involvement. For tumor response evaluation, the ability to differentiate necrotic residual mass usually seen with CT from FDG-avid tumor persistence was one of the first clinical successes of this functional imaging modality. Currently, FDG-PET/CT appears as an effective surrogate of lymphoma cell viability and is used to assess tumor response, not only at the end of treatment to decide on salvage therapy, but also during treatment with therapeutic impact to either maximize response with a more intensive regimen for slowly responding patients or reduce toxicity for early good responders and thus personalize the treatment to each patient risk. In the near feature, new PET driven metrics may be implemented in treatment decisions as new prognostic factors calculated on PET/CT images provide an interesting additional value to known prognostic parameters in several lymphoma subtypes. 

In this article, we focus on the current applications and future directions of FDG-PET/CT in managing patients with lymphoma through the development of new metabolic metrics as well as new radiopharmaceuticals.

## 2. Baseline Evaluation

FDG-PET/CT have shown better diagnostic performance than CT, especially for extra nodal involvement, as this functional imaging is able to identify tumor lesions with high glucose metabolism when no significant abnormalities are detected by CT. This led to treatment modification in several cases in which FDG-PET/CT evaluation caused upstaging of lymphoma patients.

Interestingly, FDG-PET/CT provides a better evaluation of bone marrow involvement compared to bone marrow biopsy that evaluates a very limited sample of bone marrow, whereas an FDG scan can identify focal involvement in the whole body. Invasive bone marrow biopsy is no longer indicated for the routine staging of Hodgkin lymphomas (HL) and most diffuse large B-cell lymphomas (DLBCL), and FDG-PET/CT has become the gold standard for the assessment of bone marrow invasion [3,4,5].

The baseline acquisition is also needed as reference to evaluate treatment response and is also used to calculate innovative PET driven metrics that will be described later in this review.

## 3. Interpretation Criteria Standardization and Response Evaluation Endpoints

Several interpretation criteria have been proposed to assess tumor response in lymphoma. The first FDG-PET/CT trials focused on the characterization of residual masses after end of treatment, as it was known that residual masses in CT were not strongly related to a treatment failure and thus patient’s evaluations were often classified as “Unconfirmed Complete Response” [6,7].

In 2009, in an international workshop held in Deauville (France), a standardization of these residual masses’ characterization was proposed in order to compare results across trials. This visual 5-point scale (5PS), named the Deauville score, is currently a widely used interpretation criteria in lymphoma and has even been derived to evaluate other solid cancers. The Deauville score has been reshaped in the recent Lugano classification, which introduces tumor response additionally to residual masses’ characterization but still mainly relies on Deauville score definitions. As a result, FDG-PET/CT was formally incorporated into standard staging and response assessment of FDG-avid lymphoma.

In the Lugano classification, Deauville scores 1 to 3 (residual uptake lower or equivalent to liver background) are considered as complete metabolic response (CMR), whereas 5PS scores 4 and 5 (residual uptake higher than liver background) are considered either no-metabolic-response (NMR, no change from baseline), partial metabolic response (PMR, reduced uptake compared to baseline), or progressive metabolic disease (PMD, increased uptake or new lesion). This Lugano classification has demonstrated good prognostic value and excellent inter-observer reproducibility and should now be considered as the method of choice for standardization of FDG-PET/CT reports.

Systematic surveillance exams in patients with a negative FDG-PET/CT at the end of treatment, in particular in patients with HL or DLBCL, are no longer recommended since it was found to be associated with false positives and increased radiation exposure with no evidence of benefit in patient outcome [8,9].

The recent introduction of immunomodulatory agents may complicate response assessment due to the development of a flare reaction or pseudo-progression in response to these agents. Provisional criteria have been proposed (Lymphoma Response to Immunomodulatory Therapy Criteria, LYRIC) with the introduction of the term “Indeterminate Response” to address such lesions. Renewed imaging after 12 weeks to distinguish pseudo-progression from real progressive disease is recommended [10]. A recent study from the Lymphoma Study Association (LYSA) showed that the LYRIC classification could be useful, but only for early evaluation [11]. This work found some pseudo-progression after four cycles of atezolizumab, venetoclax, and obinutuzumab (GATA trial) in relapsed or refractory (R/R) DLBCL and follicular lymphoma (FL). However, no pseudo-progression was seen at cycle 8.

All these recommendations have improved risk stratification in patients with lymphoma and result in a clearer identification of non-responders with a significant prognostic benefit. This strong clinical impact has been reported in large cohorts of patients with the most frequent lymphoma subtypes (HL, DLBCL and FL; detailed below) and is also supported by cost-effectiveness analyses [12,13].

**Figure 1:**
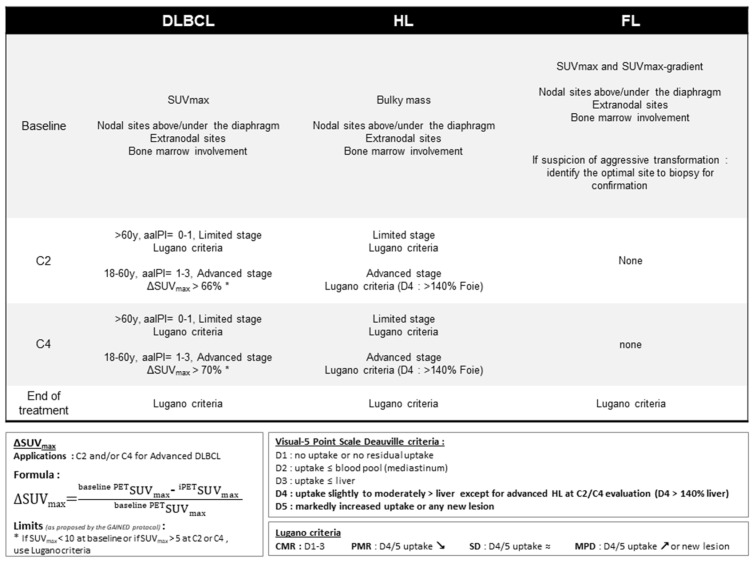
FDG-PET/CT criteria for staging and response assessment in lymphoma (aaIPI: age-adjusted International Prognostic Index; C2: after 2 cycles of chemotherapy; C4: after 4 cycles of chemotherapy; DLBCL: diffuse large B-cell lymphoma; FL: follicular lymphoma; HL: Hodgkin lymphoma; SUVmax: maximum standard uptake value).

FDG-PET/CT exams allow prediction of outcome after completion of treatment, and they also provide insights for a risk-adapted strategy to distinguish poorly responding patients requiring additional therapy, from good responders who may be exempt from part of the standard therapy, thereby limiting toxicity. The concept of interim PET (iPET) emerged in the last ten years and allows for analyzing a continuous metabolic process during treatment [14,15]. When performed after one or two cycles, iPET allows for evaluating the response of cells with the highest level of proliferation and identifying early responding chemosensitive patients without the requirement of a negative iPET at this stage. Moreover, when performed after three or four cycles, it identifies late responding patients as well as tumor regrowth (patients with iPET negative after two cycles but iPET positive after four cycles). The aim of iPET is to adapt the treatment to the intermediate response: on the one hand to de-escalate the treatment of chemosensitive patients (negative iPET) in order to limit long-term toxicity with satisfactory tumor control, and on the other hand to escalate the treatment of slow responder patients (positive iPET) and reverse their poor prognosis. Current FDG-PET/CT interpretation criteria are presented in Figure 1.

### 3.1. Advance in Hodgkin Lymphoma

The prognosis of HL has been improved with the new therapy regimens established over the last decades, including ABVD (doxorubicin, bleomycin, vinblastine, and dacarbazine) and BEACOPP (bleomycin, etoposide, doxorubicin, cyclophosphamide, vincristine, procarbazine, and prednisone) [16,17]. In parallel, significant developments occurred in the field of medical imaging at baseline and for monitoring response to therapy. FDG-PET/CT now holds a primary role in treatment planning because it evaluates the functional activity of tumor cells and appears to be much more relevant than CT, which measures the tumor size.

Two response assessment timings are now approved, including iPET during the first line of chemotherapy, and end-of-treatment FDG-PET/CT after the first line of chemotherapy (±radiotherapy).

Early treatment evaluation performed after two cycles of chemotherapy by iPET has shown a high prognostic value in several studies [15,18,19] with a high negative predictive value of 80–90% and a lower positive predictive value of 50–55%. iPET showing a residual tumor uptake after two cycles of chemotherapy (iPET2+) was associated with lower progression free and overall survival (PFS and OS, respectively) for patients treated with an ABVD or BEACOPP regimen [18,20,21], and outperformed the International Prognostic Index in patient’s prognosis identification. In this context, functional imaging with iPET has been evaluated in early and advanced stage HL in several large randomized trials including a PET-driven strategy to optimize disease control or to reduce long-term toxicity.

The intergroup randomized study H10 [22], conducted by the European Organization for Research and Treatment of Cancer (EORTC), LYSA, and Italian Lymphoma Foundation (FIL) included 1950 early stage HL patients with favorable or unfavorable risk factors according to EORTC/LYSA criteria. iPET2 negative patients received two additional cycles (in the favorable group) or four additional cycles of ABVD (in the unfavorable group) without radiotherapy. The study was interrupted because of a greater number of relapses in the without-radiotherapy strategy (6.2% vs. 2.3% in the standard arm). The final evaluation (median follow-up of 45 months) showed a significant difference in 5-year-PFS between patients exposed (99%) and not exposed to radiotherapy (87%) in the favorable group, whereas no difference was found in the unfavorable group (92% vs. 89%, respectively).

In the H10 trial [23], iPET2+ patients were randomized between two cycles of escalated BEACOPP (BEAesc) followed by involved node radiation therapy (INRT) (30 Gy) or continuing ABVD followed by INRT (30 Gy) to evaluate the benefits of a treatment escalation in iPET2+ patients. A significantly better 5y-PFS was observed in patients in the BEAesc arm compared to those pursuing ABVD cycles (91% vs. 77%). These results suggested that the treatment of patients with localized HL should be monitored by PET in routine practice to allow for treatment intensification of iPET2+ patients’ with BEAesc to enhance disease control in this subgroup of patients. This H10 trial was based on the older International Harmonization Project (IHP) PET criteria [24], originally intended for end-of-treatment assessment. The Deauville score seems to be applicable in daily practice for the intermediate evaluation of early stage HL patients as it displayed high inter-reader reproducibility and good predictive value [25,26].

The RAPID trial [27,28] included 602 HL patients with favorable disease (stages I–IIA) and no mediastinal bulk receiving three cycles of ABVD, and 571 of them were evaluated by FDG-PET/CT (iPET3). iPET3 positive patients (5PS 3–5) received one additional cycle of ABVD and 30 Gy involved field radiotherapy (IFRT). Nearly three quarters of patients (74.6%) had negative iPET3 (5PS 1 or 2) and were randomly assigned to receive 30 Gy IFRT or no further treatment. Among iPET3 negative patients, the 3y-PFS was 94.6% in the IFRT arm vs. 90.8% in the arm without radiotherapy. It should be noted that, in order to select patients presenting the most suitable profile for therapy de-escalation, the definition of PET negativity in the RAPID trial excluded Deauville score 3, which might seem to contradict the current Lugano classification. However, the final analysis of the RAPID trial did not endorse this de-escalation strategy, and PET negativity defined by Deauville score (DS1–3 vs. DS4–5) reshaped in the Lugano classification seems to be the optimal standard for therapy response evaluation in HL.

In advanced stage disease (stages IIB-IV), BEAesc in the HD18 trial [29] has demonstrated superiority in terms of PFS compared to ABVD-based therapy but without evidence of OS benefits, as non-responder patients to ABVD that had a salvage therapy showed OS similar to that of good responder patients [30]. Furthermore, this regimen showed a greater prevalence of short-term and long-term toxicities, including infertility [17], secondary leukemia, and myelodysplasia [16].

Two interesting approaches were investigated to limit BEAesc exposure. The first strategy proposed treatment escalation for poor responders after two cycles of ABVD by introducing the BEAesc. In the RATHL Study [31], after two cycles of ABVD and evaluation by FDG-PET/CT, patients with no pathological residual uptake on iPET2 (iPET2−) were randomized between 4 ABVD vs 4 AVD, and iPET2+ patients were escalated with 4 BEACOPP-14 or 3 BEACOPPesc. In the iPET2− group, no significant difference was found in the 3y-PFS between patients treated with ABVD vs. AVD (85.7% vs. 84.4%, respectively) suggesting it was unnecessary to add bleomycin for these patients. Moreover, 16% of patients presented iPET2+ and were treated by BEACOPP or BEAesc with a 3y-PFS rate of 67.5%. Similar results were observed in the Italian phase II trial showing a 62% 2y-PFS in the iPET2+ subset of patients after two cycles of ABVD receiving four cycles of BEAesc plus four cycles of baseline BEACOPP [32,33].

The second approach was a reverse strategy evaluating treatment de-escalation for good metabolic PET2− responders after two cycles of BEAesc because the negative predictive value of PET2 is significantly superior to its positive predictive value.

The AHL2011 trial [34,35] included 820 advanced stage HL patients and compared a standard arm in which patients received 6 BEAesc to an experimental arm in which patients received 2 BEAesc followed by ABVD for iPET2− patients, or four additional BEAesc cycles for iPET2+ patients. Results showed that de-escalation strategy was possible since 5y-PFS was similar in both arms (86.2% in the standard arm vs. 85.7% in the experimental arm, *p* = 0.65) with 87% of iPET2− patients in the experimental arm (vs. 88% in the standard arm) (median follow-up of 50.4 months). This demonstrated the possibility of underexposing treated patients and limiting the risks of immediate and late toxicity, confirmed by the rate of serious adverse events significantly lower in the experimental arm (72% vs. 41%, *p* < 0.00001). Furthermore, in both arms, a new time point for FDG-PET/CT decisional evaluation was investigated after four cycles (iPET4), its positivity being considered as a treatment failure and iPET4+ patients being eligible for salvage therapy. Thus, three prognostic groups were identified according to the full PET-driven strategy (iPET2/4) with a 5y-PFS of 90.9% (iPET2−, iPET4−), 75.4% (iPET2+, iPET4−), and 46.5% (iPET4+). This strategy offers an interesting stratification of the patients that is more relevant than the classical biological parameters as prognostic analysis of factors affecting PFS revealed that in multivariate analysis only the results of the complete strategy including iPET2/iPET4 remained significant. 

The HD18 trial [29] included 434 advanced stage HL patients treated with 2 BEAesc. iPET2− patients were randomized between the standard arm with six additional cycles of BEAesc or the experimental arm with two additional BEAesc. iPET2− definition only comprised Deauville scores 1 and 2 whereas Deauville score 3 was regarded as positive. iPET2− was observed in 52% patients as opposed to 87% patients in the AHL2011 trial. This difference probably came from PET positivity criteria. The AHL2011 trial used a more specific definition for positivity excluding Deauville score 3 and including Deauville 4 with a maximum residual Standardized Uptake Value (SUVmax) >140% of liver background to enhance reproducibility between readers. The Deauville score threshold used in the HD18 trial (DS1–2 vs. DS3–5) likely lacked specificity and was not pertinent for stratification of patients, as iPET2− and iPET2+ patients, with a median follow-up of 33 months, presented comparable PFS (90.8% vs. 89.7%).

### 3.2. Advance in DLBCL

During the past several years, significant prolonged survival in DLBCL has been observed in all age groups, particularly with the addition of a chimeric anti-CD20 monoclonal antibody, rituximab, to intensive cytotoxic chemotherapeutic regimens: cyclophosphamide, doxorubicin, vincristine, and prednisone (R-CHOP) [36]. Interim PET has been assessed as a potential biomarker of the early success or failure of R-CHOP in multiple studies in being able to predict those patients that are unlikely to respond to first line treatment rather than waiting for treatment to fail. As in Hodgkin’s lymphoma, it is possible to differentiate the prognosis of patients according to the response after two cycles (PET2) using the Deauville score (5PS1-3 vs. 5PS4-5). A study has also shown the benefit of evaluating the early response in 114 DLBCL patients based on the reduction in SUVmax by comparing the most hypermetabolic lesion at baseline versus the most hypermetabolic lesion on PET2 [37]. The interest of the deltaSUVmax (∆SUVmax) (fixed at 66% after two cycles) compared to the visual analysis lies in a better inter-observer reproducibility (K = 0.83 vs. 0.66, respectively) as well as a better prognostic stratification in terms of 3y-PFS (80% vs. 40% according to the (∆SUVmax); 81% vs. 59% according to Deauville Score).

The randomized phase II DLBCL LNH 2007-3B [38] including young patients (18–60 years) with unfavorable risk factors (aaIPI 2-3) investigated RCAVBP14 vs. RCHOP14 with an interim metabolic evaluation after two and four cycles. Patients who were double iPET negative (iPET2−/iPET4−) received immunochemotherapy according to their randomization arm. Slow responding patients (iPET2+/iPET4−) received intensification with ASCT. iPET4 + patients were eligible for a salvage therapy. Importantly, this PET-guided strategy reduced the number of patients undergoing intensified therapy in this population by approximately 25% compared to historical series, without adversely affecting the overall survival rate. Yet, in this study, the criteria used at this time were the IHP criteria with the same accuracy problems described in HL. The combination of iPET2 and ∆SUVmax seemed preferable than simple visual interpretation based on IHP criteria alone. Indeed, semiquantitative analysis with ΔSUVmax at iPET2 and iPET4 better predicted PFS and OS than visual analysis. The latter showed an excessive number of iPET2+ and iPET4+ results, resulting in a low predictive value for PFS and OS. Using SUVmax reduction data, 78% of iPET2+ and 80% of iPET4+ patients had a favorable 2y-PFS (77% and 83%, respectively). These good results had to be validated prospectively since the patients were not stratified on these criteria for consolidation.

The phase III prospective study GAINED [39] randomized two arms with a combination of standard chemotherapy (RACVBP14 or RCHOP14) associated with Rituximab in one arm and GA101 in the other. The same PET strategy was adopted, and consolidation was decided based on PET response. Early responders (iPET2−/iPET4−) continued with randomized chemotherapy, slow responder patients (iPET2+/iPET4−) were autografted, and non-responders after cycle 4 received salvage therapy. In this study patients were stratified on SUVmax reduction criteria. A cut-off of 66% reduction was used for iPET2 scans and a cut-off of 70% was used for iPET4 [38]. Sixty-nine percent of patients were double negative with a 2y-PFS of 90%. Slow responder patients represented 15% of all patients and did not show any difference with the first population (PFS 84.9%) after benefiting from ASCT. The third subset representing 16% of patients showed a significant lower 2y-PFS evaluated at 61.2%. Similar results were obtained in OS with no difference in the two first subsets (iPET2−/iPET4− and iPET2+/iPET4−, 2y-OS = 94.1% and 91.4%, respectively), and the third subset with a significantly lower but acceptable OS (83.1%). Conversely, another randomized controlled trial comparing different treatment approaches in aggressive B lymphoma [40,41] has used ΔSUVmax to stratify patients without success. In the PETAL study, iPET2 poor responders after a treatment with RCHOP14 were randomized between continuation of RCHOP vs. a Burkitt-like protocol (B-ALL protocol). The results showed that patients’ outcome was not modified according to the randomized treatment. In particular, the Burkitt-like intensification did not make it possible to reverse the pejorative prognostic value of iPET2+.

Recently an individual patient data meta-analysis was built to determine the optimal timing and optimal PET positivity criteria for interim PET to predict response in 1977 DLBCL patients from the PETRA database [42]. All patients had an interim PET following one to four cycles of therapy. A cut-off of 66% reduction was used for PET scans after one, two, or three cycles and a cut-off of 70% was used for iPET after four cycles. Only iPET2 and iPET4 were able to significantly discriminate responders from non-responders with higher hazard ratios (HR) for iPET4 (HR = 2.36 and 3.67 for iPET2 and iPET4, respectively). Regarding iPET negative patients, there was no significant difference in PFS using any of the response criteria at the four assessed time points. iPET2, thus appeared as the optimal timing to identify responders, as there was no significant increase in survival at later times, regardless of PET criteria, whereas iPET4 might be the optimal timing to identify non-responders. These data confirmed the potential role of iPET in the design of response-adapted trials. Nevertheless, efforts should be made to improve the strategy with the use of adapted and reproducible positivity and interpretation criteria in order to optimize the efficacy/tolerance ratio of the treatment.

Despite the fact that FDG-PET/CT is accepted as the current gold standard for response assessment, 15–20% of DLBCL patients with metabolic CR will experience disease recurrence. The exploration of additional response assessment approaches is justified. Growing evidence is emerging on the value of next-generation sequencing techniques to identify minimal residual disease and circulating tumor cell DNA to assess response [43]. FDG PET/CT imaging has transformed the way lymphoma patients are managed. It is now regarded as key to accurate staging and has been adopted as the basis for broadly accepted response criteria. The inclusion of molecular genetic and biomarker studies could enhance the sensitivity and specificity of FDG-PET/CT, increase its negative and positive predictive values, and strengthen the role of this major imaging technique in the care of lymphoma patients.

### 3.3. Advance in FL

FL represents the most common indolent lymphoma and can have a variety of clinical presentations [44]. About 95% of all FL present with FDG avidity. Thereby, FDG-PET/CT showed better diagnostic performance than CT since it upstaged approximately 10–60% of patients with early stages to advanced stages [45,46], contributing to improvement in PFS. Furthermore, it could be useful to more precisely identify patients eligible for curative treatment and guide biopsy for diagnostic confirmation or suspicion of transformation. However, bone marrow biopsy is still recommended as the gold standard for diagnosis of bone marrow invasion.

Regarding response assessment, the Deauville Score analysis of post-induction FDG-PET/CT seemed to represent, in this histologic subtype also, a helpful prognostic tool. It better identifies patients with no active metabolic disease (complete metabolic responses) compared to the CT-based or IHP response evaluation criteria [47] with improved PFS and OS.

### 3.4. Advance in Mantle Cell Lymphoma

Mantle Cell Lymphoma (MCL) is an aggressive subtype of NHL that represents about 5% of all NHLs [48]. The value of FDG-PET/CT evaluation in MCL has not been extensively studied up to now. Nevertheless, its value at baseline showed a good sensitivity for initial staging [49]. Moreover, several works studied the possible benefit of early and end-of-treatment metabolic assessment in MCL, although these findings must be confirmed and validated through prospective studies. In this context, metabolic information could be considered in the MCL treatment strategy in order to identify good and poor patients that may benefit from more aggressive treatment.

The LyMa-PET study [50,51] issued from the LyMa trial evaluating the predictive value of FDG-PET/CT at diagnosis in untreated MCL patients, highlighted a SUVmax cut off (>10.3) associated with poorer PFS (*p* = 0.0003) and OS (*p* = 0.0003). SUVmax appears to have an even greater prognostic value when combined with clinical and biological biomarkers [52]. Indeed, IPI score combined to SUVmax allowed classifying patients with MCL into distinct risk categories with different PFS length: low (29%; no relapse/progression), intermediate (42%; median PFS: 37 months) and high risk (29%, median PFS: 22 months). These observations were also found in the LyMa-PET study [51]. The identification of patients at very high risk of early progression after first line therapy should allow FDG-PET/CT to be definitely integrated into the treatment strategy in order to improve the management of the disease.

## 4. Metabolic Evaluation in the Immunomodulatory Therapy Era

FDG-PET also plays an essential part in the management of patients resistant to chemotherapy that may benefit from therapies with different mechanisms of action. Immunotherapy, relying on enhancing the immune response to the tumor, is a highly attractive approach for the management of many tumor types. As described above, advances in immunomodulatory treatments that impact on the interpretation of imaging have led to the need to revise criteria for staging and response. The LYRIC criteria, proposed by Cheson et al., introduced a new response category, Indeterminate Response. This approach integrates possible pseudoprogression [53] described with checkpoint inhibitors and also immune modulators in general, in order to avoid stopping a therapy that is actually effective, and mandates additional biopsies or reimaging after 12 weeks.

In addition, studies with immune checkpoint inhibitors and bi-specific T-cell engagers have shown the promise of T-cells in the treatment of cancer. To be effective, T-cells must have the right specificity for a tumor, be available in adequate amounts, and overwhelm any local immunosuppressive environment. Chimeric antigen receptor (CAR) engineered T-cells may address these issues and have generated considerable expectations. Axicabtagene ciloleucel (axi-cel) and tisangenlecleucel (tisa-cel) are engineered autologous T-cells for which a subject’s individual T-cells are harvested and genetically modified to target CD19 expressed on the surface of B-cell lymphomas. In August 2018, axi-cel and tisa-cel were given approval in Europe for R/R DLBCL and transformed FL (trFL) after two or more lines of systemic therapy. These approvals were supported by two large trials [54,55,56]. In the JULIET trial, Tisa-cel resulted in increased overall response rate (ORR) of 52%, with a 40% CR rate and median OS of 12 months. In the ZUMA-1 trial, Axi-cel led to an 83% ORR, a 58% CR rate, and a median OS of 25.6 months. In these studies, Lugano criteria were used and after CAR T-cell therapy, CR of FDG-avid lesions can be as long as 9–12 months.

## 5. New Metrics

### 5.1. Total Metabolic Tumor Volume

In the last few years, TMTV has been proposed as a new prognostic parameter in various lymphoma subtypes.

TMTV is a quantification of the whole-body tumor burden using FDG-PET/CT images. This quantification relies on a segmentation of each tumor uptake in the full body acquisitions [57,58].

Initially explored in retrospective series, this new quantification has been validated in ancillary studies of clinical trials and may be implemented in prospective trials to tailor risk adapted treatment strategy.

#### 5.1.1. HL

Tumor burden quantification in HL has been done for decades and such quantification has been historically based on clinical evaluation and planar radiography in the 1990s and on computed tomography starting in 2000 [59,60].

As functional imaging provides better sensitivity in tumor staging, it provides a better tumor burden quantification, especially regarding the extra nodal involvement compared to conventional imaging.

Thus, several papers have now assessed the clinical prognostic value of TMTV in HL.

For early stage HL, a TMTV over 147 mL has been shown to be prognostic of PFS and OS and, interestingly, this prognostic value is additional to the interim response evaluation at two cycles identifying a subgroup of patient having initial high tumor burden and partial response at two cycles with a very low outcome (5y-PFS 25%) [61].

This prognostic value in early stage has also been shown in another retrospective series of 267 patients and has been proposed as a parameter to reclassify early GHSG unfavorable group as the TMTV split this population in subgroups having a survival similar to that of favorable early stage group for TMTV < 268 mL and to advanced HL for TMTV > 268 mL [62].

In advanced HL, data are still insufficient. In an ancillary study of the HD18 trial, TMTV was predictive of PET2 positivity but was not statistically significant for survival prediction. However, this study suffers from the very limited number of events (16 events) in this series [63].

The TMTV analysis of the AHL2011 showed promising results for the interim survival analysis but it is still awaiting validation in the final follow up analysis [64].

Finally, prognostic interest has also been shown in relapsed HL and provides an additional prognostic value complementary to PET response evaluation before autologous stem cell transplantation [65]. Thus, TMTV calculation at relapse could also be used to tailor salvage therapy strategies in relapsed HL.

#### 5.1.2. DLBCL

In DLBCL, baseline TMTV has been proposed as a prognostic factor in various studies [66] and recently confirmed by the analysis of the PETAL trial, showing that baseline TMTV over 328 mL was predictive of a poorer outcome and this prognostic value is additional to the response evaluation using ∆SUVmax criteria. The combination of both baseline TMTV and ∆SUVmax, similarly to Hodgkin Lymphoma, identifies three prognostic subgroups of patients with PFS ranging from 91% (low TMTV, complete response) to 30% (high TMTV, no complete response) [67].

The combination of TMTV and tumor gene expression has also shown interesting prognostic value by allowing patient risk stratification additionally to the GCB/ABC phenotype [68,69].

TMTV has been reported to influence rituximab exposure during first line therapy in a pharmacological study and thus could be used to adjust the therapeutic index by using a personalized dose of rituximab [70].

Interestingly, TMTV seems to represent a promising prognostic tool, as a high tumor volume may correlate with a more severe cytokine release syndrome [71,72]. However, further explorations are needed to confirm these findings.

#### 5.1.3. FL

TMTV showed a prognostic value when assessed before first line therapy of FL since patients with a TMTV > 510 mL have a lower PFS. In FL, TMTV was reported to be moderately correlated (r = 0.6) with circulating tumor and cell free DNA, showing different prognostic values. This suggests that these two tumor burden quantifications may be used in combination as a new prognostic tool [73].

#### 5.1.4. Issues to Solve

To implement TMTV in therapeutic decisions several issues are still to be solved.

Despite a large number of clinical studies with concordant conclusion in TMTV prognostic value, the TMTV cut off value to distinguish patients has significant variability. 

This is probably due to different criteria in patient selection and different methodologies to segment PET/CT images as several segmentation thresholds have been proposed with a clear impact on TMTV values [57,74,75,76]. A consensual segmentation methodology is still pending.

The second issue to solve is the automatization of TMTV calculation. This automatization has been dramatically improved thanks to new deep learning algorithms that are now showing very good accuracy in reproducing user manual segmentation [77,78]. These algorithms still need a comparative approach to select the best method to be implemented in a medical viewer to provide a valuable automatic segmentation that will reduce the variability of the manual delineation of TMTV and thus improve reproducibility and acceptability of this whole-body segmentation for nuclear medicine physicians.

Lastly, the clinical value of TMTV derived parameters is still to be evaluated, notably TLG (Total Lesion Glycolysis), which is the product of TMTV and SUVmean, as reported in some studies. This index is highly correlated with TMTV and its prognostic strength may be more specific to some lymphoma subgroups.

### 5.2. Radiomics

“Radiomics” is a generic word for medical image measurement used as image descriptor and prognostic tool. By that definition, TMTV or simple SUVmax measurement could be considered as “Radiomics”. However this term often refers to more sophisticated image calculation such as shape or textural analysis [79].

Radiomic analysis results in thousands of possible quantifications parameters (a significant part of them are correlated to each other) [57,80,81].

#### 5.2.1. Textural Approaches

A common quantification in the radiomics field is textural analysis. This approach quantifies tumor distribution of uptake values, either at a local level (pixel by pixel or voxel by voxel) or regional (groups of pixels/voxels of near intensity). Hundreds of parameters could be calculated from these approaches and thus results are not easy to interpret or to reproduce.

However, this textural approach has shown capabilities to identify patients prognosis in DLBCL [82,83] or mantle lymphoma [84,85] or to constitute a diagnostic parameter for the detection of bone marrow involvement [86].

#### 5.2.2. Whole Body Tumor Geometry Approaches

Another radiomics approach is to quantify tumor distribution spatially in the body with quantification indexes such as spread distance and fragmentation.

The tumor spread calculation could be calculated easily by computing the maximal distance between tumoral uptakes. This parameter called Dmax has been shown to be additionally prognostic to TMTV in quantifying the ability of the tumor to spread independently from its mass [87]. This parameter in combination with TMTV allows identifying new prognostic subgroups.

Another approach proposed is to calculate tumor fragmentation. This could be done by calculating the surface to volume ratio. If the tumor burden is fragmented in multiple small uptakes it will expose a higher surface compared to the total metabolic tumor volume and this constitutes a quantification of the tumor/host interface that could influence therapeutic response in many ways and has shown interesting prognostic value [88].

#### 5.2.3. Remaining Issues for Radiomics Parameters

As with TMTV, these radiomics approaches are still lacking standardization. 

First of all, radiomics quantifications rely on initial tumor segmentation, and thus are affected by the same issues presented earlier in TMTV quantifications (lack of consensual segmentation methodology, inter-reader reproducibility, time consumption).

Additionally, described parameters had historically non-consensual implementation, which has now significantly improved thanks to Image Biomarker Standardisation Initiative (ISBI) editing references for radiomics terms and calculations that are now available in several software packages, providing reproducible radiomics quantifications [89].

Finally, due to the high amount of available quantifications, results are difficult to interpret and sometimes difficult to link to a physiopathological process. However future clinical data should make it possible to select some robust and valuable parameters that could be included to tailor risk personalized strategies.

## 6. PET Tracers in Lymphoma beyond FDG

**Figure 2:**
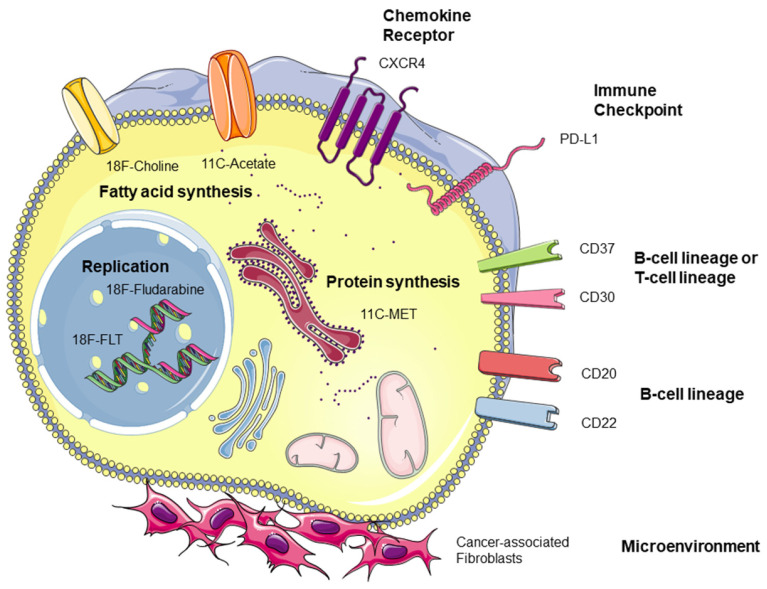
Receptors and metabolic pathways for molecular imaging applications in lymphoma.

As described above, FDG remains today the leading PET tracer for routine molecular imaging in haematology and oncology, and its benefits and limits are extensively reported. Yet, while FDG-PET/CT has quickly found its place in the diagnostic, prognostic, and therapeutic evaluation of patients with lymphoma, it is among patients with solid tumors that other tracers are most successful today. Indeed, molecular imaging of lymphomas, beyond the context of radioimmunotherapy, has not been fully exploited while there is undoubtedly a demand for more precise probes targeting other metabolic pathways or specific receptors in lymphomas. Main targets are presented in Figure 2. Among others, the tendency towards complex engineered therapies for haematological malignancies relying on phenotypic or genetic tumor characteristics provides great opportunities for molecular imaging in therapeutic management.

### 6.1. Explorations of Other Metabolic Pathways

The use of 18F-Fluorothymidine (FLT) and 11C-Methionine (MET), amino-acid tracers whose uptake indirectly reflects cellular proliferation, was reported. Preliminary results with MET in children and young adults with HL demonstrated limited results [90], as opposed to studies with FLT showing a good correlation with lymphoma lesions and a sensitivity at baseline similar to FDG [91]. A higher specificity of FLT uptake especially in regards to post therapeutic inflammation suggested improved assessment of treatment response and prediction of outcome [92,93,94,95,96,97]. Larger studies on the usefulness of FLT-PET/CT in the evaluation of lymphoma patients with FDG-avid residual masses at the end of treatment may be worth considering.

Based on fludarabine, a drug already used in low grade lymphomas, 18F-Fludarabine (2-[18F] fluoro-9-β-D-arabinofuranosyl-adenine) also appears as a good candidate to enhance diagnostic accuracy and therapeutic evaluation especially in low or heterogeneous FDG-avid lymphoma, showing high selectivity for lymphoid cells, regardless of the cell cycle [98,99,100,101]. Pre-clinical studies and first-in-humans reports [101], in DLBCL and chronic lymphoid leukaemia patients, demonstrated lower uptake in inflammatory cells compared to FDG and a better correlation with histology than the latter. Good sensitivity of 18F-Fludarabine for detection of indolent lymphomas lesions was reported [101] without uptake on inflammatory lesions, thus avoiding false-positive results. Further explorations are warranted and an exploratory, multicenter prospective clinical trial for initial staging and therapeutic evaluation in DLBCL, HL, and FL is ongoing.

In addition to their widespread use in solid cancers, the performance of lipid tracers also looks promising in lymphoma, although the literature is currently more limited. Tsuchiya et al. have compared the value of 11C-acetate and FDG-PET/CT in a small series of patients [102] with apparent greater sensitivity of 11C-acetate in the detection of indolent lymphomas. The performance of 18F-Choline is only reported through accidental findings [103,104,105]. These results need to be verified in larger homogeneous populations.

### 6.2. Phenotypic Imaging

The spread of phenotypic PET imaging using monoclonal antibodies (mAbs) or peptides provides a set of non-invasive options to investigate in vivo targets’ expression and distribution and to acquire robust diagnostic, prognostic, and theranostic information. So far, in lymphoma, the vast majority of studies focusing on phenotypic imaging have evaluated in the context of radioimmunotherapy for mAbs distribution, pharmokinetics, and absorbed radiation doses [106,107] targeting, among others, CD20, CD22, CD30, or CD37. Moreover, beyond the numerous preclinical studies, most of them investigated SPECT/CT imaging that provides lower spatial resolution and specificity. Therefore, despite the potential of molecular imaging for the acquisition of target-specific information in the context of immunomodulatory therapy and personalized medicine [108,109,110], it has not played an important role in lymphoma to date. However, a few studies, albeit in small and heterogeneous populations, have reported interesting results, especially with CD20, the most frequently studied target, including the importance of preloading on the biodistribution of tracers [111], the exploration of CD20 expression in patients having relapsed after rituximab treatment [111,112], or even the confirmation of the absence of benefit of this therapy in primary central nervous system lymphoma patients [113].

A novel and promising target is CXC chemokine receptor 4 (CXCR4). This transmembrane receptor is involved in the cell migration process and in the homing of hematopoietic stem cells to the bone marrow compartment. Several studies reported the use of 68Ga-Pentixafor that showed a high contrast in CXCR4-expressing lymphomas [114,115,116,117]. Furthermore, for a theranostic approach, its therapeutic properties are currently under investigation, radiolabeled with β- or α-emitters [118]. Preliminary results are encouraging with good tolerance of the treatment and promising initial response rates in NHL patients.

Fibroblast activation protein inhibitor (FAPI) also emerges as a new and highly promising probe for diagnostic and possibly theranostic application in various neoplasms including lymphoma. FAP is overexpressed by cancer-associated fibroblasts (CAFs) present in tumor microenvironment, which provides a high tumor uptake and a very low accumulation in normal tissues, resulting in excellent signal-to-noise ratios [119,120].

Finally, another area for future development of PET imaging in lymphoma is the rise of immunomodulatory therapy options, such as immune checkpoint inhibitors and CAR T-cells therapy. Indeed, despite the lack of literature in haematological neoplasia, these applications generate many expectations in view of the importance of these two therapeutic options in NHL. In this way, a recent study reported the feasibility of PET imaging with radiolabeled programmed cell death-ligand 1 (PD-L1) mAbs for the assessment of therapy in solid malignancies [121].Huge challenges remain in monitoring response to CAR-T cells therapy. A potential solution might be the tracking of CAR-T cells [122,123]. Molecular imaging, through visualization of the biodistribution of CAR-T cells, may offer unique information on the targeting of lymphoma lesions. In addition, CD19 imaging could be employed to identify patients presenting loss of expression of CD19, a key mechanism of resistance for patients receiving this therapy.

## 7. Conclusions

FDG-PET/CT stands as the main imaging modality in lymphoma. Compared to computed tomography, FDG-PET/CT improves staging and end of treatment evaluation, including improved residual mass evaluation by discriminating between fibrosis and remaining disease.

Early assessment of response to therapy demonstrated strong prognostic value, and a series of recent prospective randomized phase III studies confirmed the early PET-driven therapeutic strategy, leading the way to a novel area of personalized medicine, optimizing disease control and toxicity.

New FDG-PET/CT parameters are being developed to assess response to new immunotherapy regimens and provide an improved prognostic factor for predicting patients’ survival. Radiomics in particular is a very challenging area of research. In contrast to histological biopsy-based invasive biomarkers that examine a limited area of tumor, radiomics non-invasively investigates the entire lesion or disease. Because visualizing tumor heterogeneity is crucial for assessing the aggressiveness and prognosis of lymphoma, radiomics might offer tremendous possibilities in the management of these patients.

Finally, the identification of pathways or specific receptors in lymphomas has enabled the development of new radiopharmaceuticals providing great opportunities for molecular imaging in treatment evaluation and management. It is unclear whether these radionuclide probes besides FDG will have a part to play in clinical routine diagnostics. However, the tendency for very advanced treatments, which depend on the genetic and phenotypic composition of lymphoma cells, provides exciting opportunities for nuclear medicine in the context of immunotherapy and personalized medicine. Ultimately, molecular imaging could lead to greater cost-effectiveness by allowing candidate selection for expensive targeted therapies, among which CXCR4 is a serious candidate. Other new tracers, such as FAPI, could be used in addition to ¹FDG-PET/CT imaging, specifically in indolent lymphoma patients.
